# Measles Elimination Efforts and 2008–2011 Outbreak, France

**DOI:** 10.3201/eid1903.121360

**Published:** 2013-03

**Authors:** Denise Antona, Daniel Lévy-Bruhl, Claire Baudon, François Freymuth, Mathieu Lamy, Catherine Maine, Daniel Floret, Isabelle Parent du Chatelet

**Affiliations:** Author affiliations: Institut de Veille Sanitaire, Saint-Maurice, France (D. Antona, D. Lévy-Bruhl, C. Baudon, M. Lamy, C. Maine, I. Parent du Chatelet);; National Reference Centre for Measles and Respiratory Paramyxoviridae, CHU Caen, France (F. Freymuth);; Université Claude Bernard Lyon 1, Lyon, France (D. Floret)

**Keywords:** measles, France, epidemiology, elimination, viruses, vaccines, immunization, children, adolescents, young adults, *Suggested citation for this article*: Antona D, Lévy-Bruhl D, Baudon C, Freymuth F, Lamy M, Maine C, et al. Measles elimination efforts and 2008–2011 outbreak, France. Emerg Infect Dis [Internet]. 2013 Mar [*date cited*]. http://dx.doi.org/10.3201/eid1903.121360

## Abstract

Although few measles cases were reported in France during 2006 and 2007, suggesting the country might have been close to eliminating the disease, a dramatic outbreak of >20,000 cases occurred during 2008–2011. Adolescents and young adults accounted for more than half of cases; median patient age increased from 12 to 16 years during the outbreak. The highest incidence rate was observed in children <1 year of age, reaching 135 cases/100,000 infants during the last epidemic wave. Almost 5,000 patients were hospitalized, including 1,023 for severe pneumonia and 27 for encephalitis/myelitis; 10 patients died. More than 80% of the cases during this period occurred in unvaccinated persons, reflecting heterogeneous vaccination coverage, where pockets of susceptible persons still remain. Although vaccine coverage among children improved, convincing susceptible young adults to get vaccinated remains a critical issue if the target to eliminate the disease by 2015 is to be met.

In 1983, measles vaccination was introduced into the immunization schedule for toddlers in France; the combined measles-mumps-rubella vaccine (MMR) has been used since 1986. A second MMR dose was added in 1996. Until 2004, recommendation were that the first dose (MMR1) be administered at 12 months of age and the second (MMR2) at 3–6 years of age. A catch-up schedule with 1 dose of MMR was also recommended for unvaccinated children 6–13 years of age.

To meet the World Health Organization (WHO) European Region’s goals for measles elimination, a national plan was implemented in 2005. It included bringing forward the administration of MMR2 to a child’s second year of life in addition to expanding catch-up to include 2 doses for unvaccinated persons born after 1991 and 1 dose for those born during 1980–1991. Other measures implemented included the vaccination of susceptible health professionals and detailed control measures around suspected cases (*1*,*2*).

Measles was a notifiable disease from 1945 to 1986 in France. From 1986 to 2004, surveillance of the disease was managed through a national sentinel network of general practitioners (*3*). Because cases were becoming rare, mandatory reporting was reintroduced in 2005. Laboratory confirmation by serologic or saliva testing and including virus characterization was simultaneously implemented.

Only 40 and 44 cases were notified in 2006 and 2007, respectively, which placed the incidence of reported cases below the WHO threshold for measles elimination (0.1 cases/100,000 inhabitants). However, in 2008, the number of cases started increasing and rose dramatically thereafter (*4*,*5*). Here, we describe the 2008–2011 measles epidemic in France and the characteristics of cases reported over that period.

## Vaccine Coverage 

Vaccine coverage is measured through the analysis of infants’ health certificates, which are filled in during a mandatory medical examination when children reach their second birthday. The certificates are sent by physicians to the district Maternal and Child Health Offices, then aggregated at a national level and analyzed by the Institut de Veille Sanitaire (InVS). Coverage in older children is assessed through random sampling school surveys conducted among children 6, 11, and 15 years of age (*6*).

**Figure 1:**
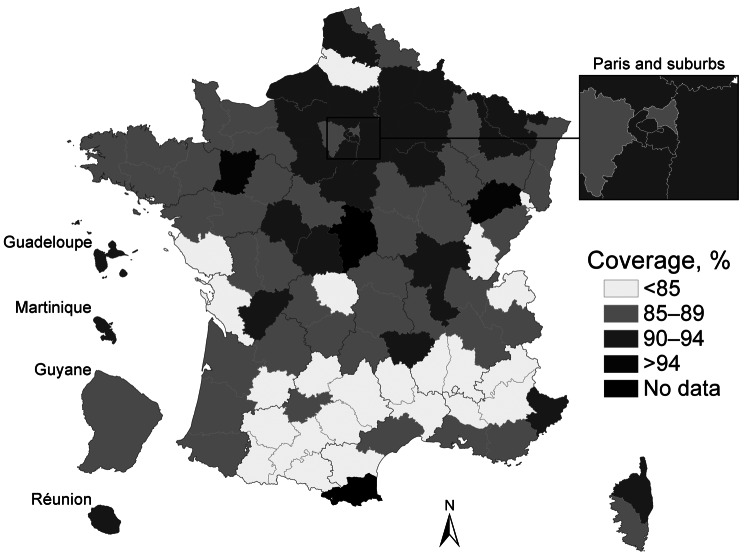
Coverage of initial measles-mumps-rubella vaccination (MMR1) listed in health certificates for children at 24 months of age, by district (département), France, 2003–2008. Data are latest available figures for the period. Sources: Institut de Veille Sanitaire, Ministry of Health statistical department.

**Figure 2:**
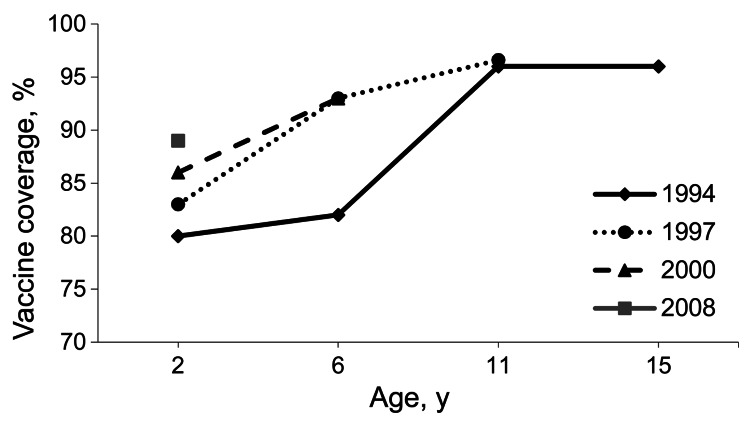
Measles vaccine coverage for 4 birth cohort years, France. Sources: Institut de Veille Sanitaire, Ministry of Health statistical department, Ministry of Education.

The MMR1 coverage at 2 years of age increased steadily in the 1980s, leveling off at 80%–85% of the target population in the 1990s. Although coverage has improved since then, it never exceeded 89%–90% in children born between 2005 and 2008. Furthermore, differences in coverage persist between regions, with many districts in southern France still below 85% (Figure 1). Since 2002, the MMR1 coverage in school children has remained consistent at between 93% and 96%, reflecting a significant proportion of catch-up vaccinations being administered to children >2 years of age. Figure 2 shows the increase over time of MMR1 coverage for different birth cohorts. In 2008, the MMR1 coverage reached 96.6% in children 11 years of age.

The same sources were used to monitor the vaccine coverage for MMR2. In 2-year-old children, the MMR2 coverage improved over time, with a 2-fold increase (from 29.3% to 60.9%) between 2006 and 2010. In older children, results from school-based surveys (www.invs.sante.fr/Dossiers-thematiques/Maladies-infectieuses/Maladies-a-prevention-vaccinale/Couverture-vaccinale/Donnees) showed a significant increase over time: from 28.1% in 2003 to 45.1% in 2006 in 6-year-old children; from 56.8% in 2002 to 74.2% in 2005 and to 85.0% in 2008 in 11-year-old children. In 15-year-old children, the MMR2 coverage increased from 65.7% in 2004 to 84.0% in 2009 (provisional data for 2008 and 2009). No vaccine coverage data are available for persons >15 years of age.

## Epidemiology of Measles Outbreak

To describe the epidemic, we included cases notified from January 1, 2008, through December 31, 2011, that fulfilled the criteria for reporting. Clinicians and biologists notify cases to the Regional Health Agencies responsible for implementing control measures and sending notification forms to the InVS (notification forms and case definitions available online, www.invs.sante.fr/Dossiers-thematiques/Maladies-infectieuses/Maladies-a-prevention-vaccinale/Rougeole). Cases were classified as clinical or confirmed (biologically or epidemiologically) as described (*4*). Case patients without any known exposure to a measles case in France and who had been in a measles-endemic country 7–18 days before the rash onset were considered imported.

Population estimates from the National Institute of Statistics and Economic Studies were used to calculate incidence rates. Proportions were compared by using the χ^2^ test. The χ^2^ for trend was used to test associations between age groups and types of complications in hospitalized patients. Descriptive analysis was done by using Stata version 11 software (StataCorp LP, College Station, Texas, USA).

The date of the appearance of the rash was used as the disease onset date. Epidemic waves were defined as cases occurring between October and the following September over the 4-year period, resulting in the identification of 3 waves. Notifications to the national nosocomial surveillance system were also included in the analysis.

During spring 2008, several measles clusters were identified among students at private schools operated by a traditionalist religious group; secondary household clusters also occurred (*4*). Ministry of Health (MOH) representatives contacted the group’s leaders, but these discussions were unsuccessful in mitigating parents’ reluctance to have their children vaccinated. The virus progressively spread out of this community; subsequent outbreaks were reported in both private and public schools. In 2009, community-wide transmission was established.

From January 2008 through December 2011, a total of 22,686 measles cases were notified. We excluded from analysis 84 cases in which postvaccination rash was reported (defined clinically as rash occurring 5–20 days after vaccination in the absence of any known exposure to a measles case); for 15 (18%) patients with known or possible contact with a case, a saliva sample was sent to the National Reference Center (NRC) for virus genotyping, and the vaccine virus was identified. We also excluded 399 cases with negative tests and 29 nonresident patients exposed to measles during a temporary stay in France. In total, 22,178 cases were included in the analysis, 447 of which were imported (including 230 from Europe).

### Spatiotemporal Evolution of the Epidemic

**Figure 3:**
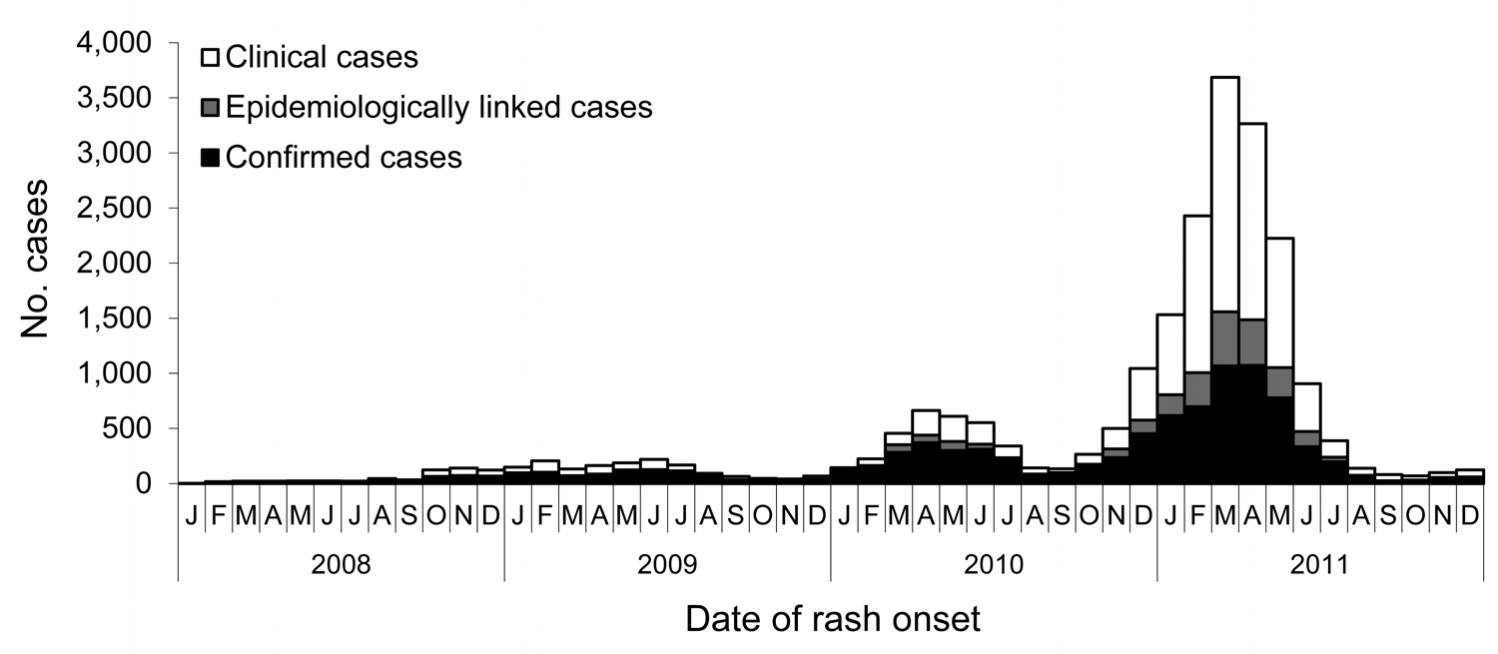
Number of notified measles cases per month, determined by date of rash onset, France, January 2008–December 2011.

Among the 22,178 cases we analyzed, 603 were reported in 2008, 1,543 in 2009, 5,083 in 2010, and 14,949 in 2011. The epidemic curve (Figure 3) showed that the number of cases started increasing in mid-2008, evolving in 3 epidemic waves. A total of 21,669 cases were reported from October 2008 through September 2011: 1,774 during the first wave, October 2008–September 2009; 3,429 during the second wave, October 2009–September 2010; and 16,466 during the third wave, October 2010–September 2011. Incidence of measles during the study period was 2.7 cases per 100,000 inhabitants during the first wave, 5.2/100,000 during the second wave, and 25.6/100,000 during the third wave. Peaks were observed in April 2010 (659 cases) and March 2011 (3,642 cases).

**Figure 4:**
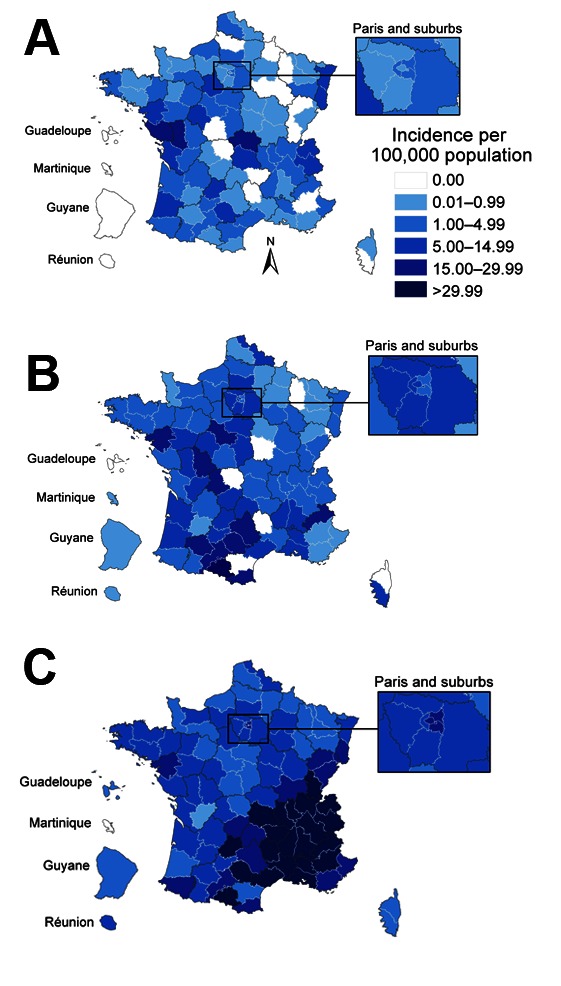
Evolution of geographic distribution of measles cases during 3 epidemic waves, France. A) October 2008–September 2009; B) October 2009–September 2010; C) October 2010–September 2011.

The geographic distribution of cases was analyzed for the 21,240 (96%) patients who had a documented place of residence. Among them, 14 had been exposed to measles in mainland France but lived in French overseas districts: La Réunion (6), French Guyana (1), Guadeloupe (5), or Martinique (2). The virus circulated nationwide, but southern France was the area most affected, especially during the third wave; incidences in the Rhône-Alpes, Provence-Alpes-Côte d’Azur, and Languedoc-Roussillon regions reached 97.2, 53.9, and 48.3 cases/100,000 persons, respectively (Figure 4).

## Case Characteristics

### Case Classification and Patient Sex and Age

Of the 22,178 cases analyzed, 10,711 (48.3%) met the clinical case definition, 8,847 (39.9%) were confirmed biologically, and 2,620 (11.8%) were linked epidemiologically to a biologically confirmed case. The male-female ratio for patients was 1.05 and was comparable across all age groups and epidemic waves.

Age was known for 22,087 (99.6%) patients. Median age increased over time: 12 years during the first wave (interquartile range [IQR] 5–18 years), 14.5 years during the second wave (IQR 4–24 years), and 16 years during the third wave (IQR 7–24 years). During the third wave, incidence reached 134.6 cases/100,000 in infants <1 year of age, 68.6 cases/100,000 in children 10–19 years of age, and 46.8 cases/100,000 in persons 20–29 years of age. 

**Figure 5:**
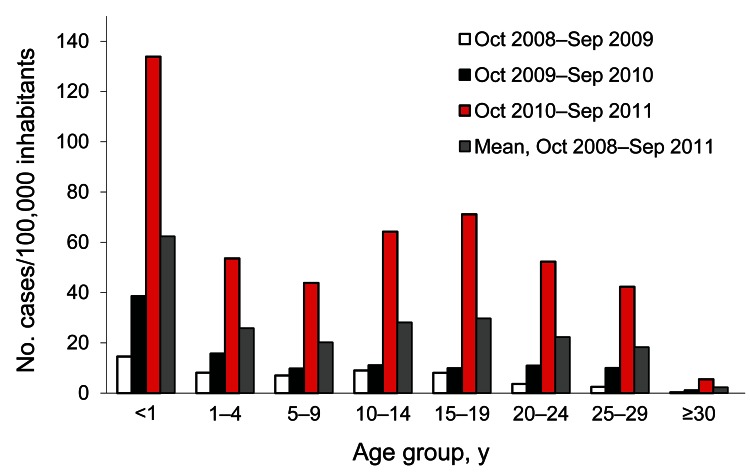
Incidence of measles cases during 3 epidemic waves, by patient age group, France, 2008–2011.

Infants <1 year of age were the most affected by the increasing number of cases between waves. Incidence for this age group was 2.6× higher for the second wave compared with the first and 9.8× higher for the third wave compared with the first. Of 1,572 measles cases reported in this age group (7.5% of all cases), 29 were in infants <1 month of age, including 13 neonatal cases; 269 were in infants 1–5 months of age, 547 in infants 6–8 months of age, and 727 in infants 9–11 months of age. However, increases among all age groups were substantial; from the first to the third waves, incidence increased 7-fold for those 1–9 years of age (from 8.4 to 54.1 cases/100,000 children), 8-fold for those 10–19 years of age (from 8.6 to 68.3 cases/100,000 children), and 15-fold for those 20–29 years of age (from 3.1 to 48.1 cases/100,000 persons) (Figure 5).

### Vaccination Status

Patient vaccination status was reported by clinicians for 18,434 (83%) cases; for 6,841 (37.1%) patients, status was verified by a vaccination document listing the date of the last injection. Among these 6,841 patients, 1,375 (20.1%) were vaccinated, 1,041 (15.2%) with 1 dose and 318 (4.7%) with 2 doses. Data on the number of doses were not available for 16 patients (0.2%).

**Figure 6:**
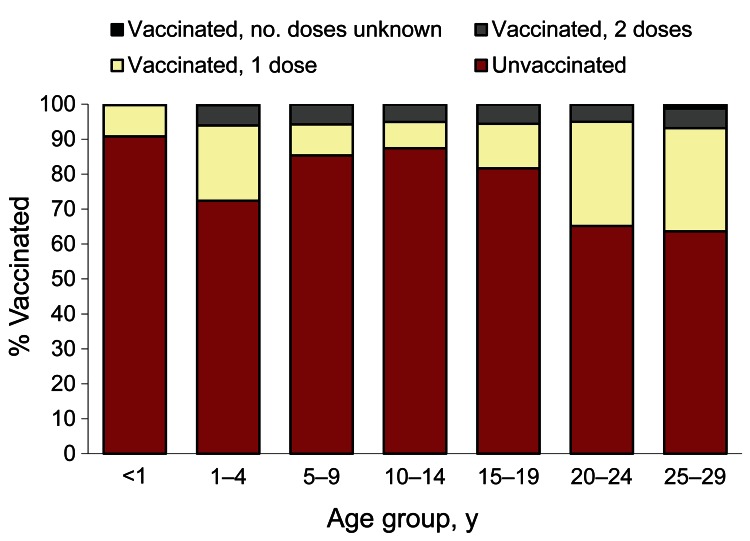
Vaccination status of measles patients, by age, France, January 2008–December 2011. Vaccination status was unknown for 80 patients.

The proportion of vaccinated cases differed significantly between age groups (p<0.001). Of persons 20–24 years of age, only 34.8% had been vaccinated: 26.9% with only 1 dose, 4.8% with 2 doses, and 0.1% with an unknown number of doses (Figure 6).

### Case Severity

During the 4-year study period, 2,582 (11.6%) measles cases involved complications; most frequently reported were pneumonia (1,375 cases, 6.2%), acute otitis media (321 cases, 1.4%), and hepatitis or pancreatitis (248 cases, 1.1%). Diarrhea was reported in 100 cases (0.4%).

**Figure 7:**
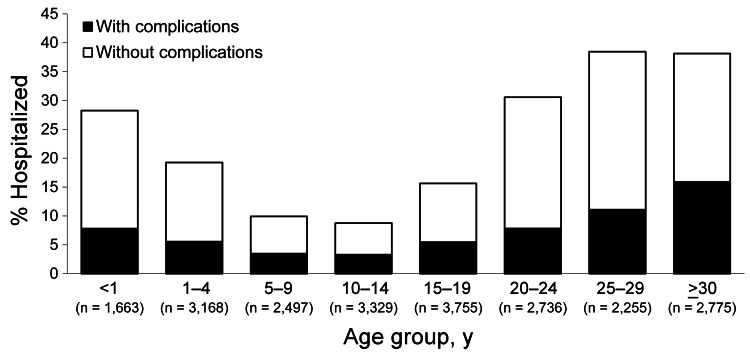
Percentage of measles patients hospitalized, with and without reported complications, by age group, France, January 2008–December 2011.

Overall, 4,980 (22.4%) measles patients were hospitalized, with substantial differences in hospitalization rates between age groups. Hospitalization rates were 28% for infants <1 year of age and 31%–38% for adults (Figure 7). Among hospitalized patients, the most frequently reported complication was pneumonia (1,023 cases, 20.6%) (Table). The male-female ratio (1.0) for patients with complications was similar to that for patients without complications; median patient age was 24 years (IQR 11–32 years). The proportion of pneumonia cases increased with age, reaching 28.8% in adults >30 years of age (p<0.001 for comparison of proportion of pneumonia in adults and overall rate of pneumonia).

**Table:** Measles-related complications among 4,968 hospitalized patients, by age group, France, January 2008–December 2011*

Complications	No. (%) patients, by age group, y					p value†
	<1, n = 470	1–14, n = 1,150	15–29, n = 2,290	>30, n = 1,058	Total, n = 4,968	
All complications	130 (27.7)	373 (32.4)	669 (29.2)	441 (41.7)	1,613 (32.5)	<0.001
Pulmonary						
Pneumonia	75 (15.9)	227 (19.7)	416 (18.2)	305 (28.8)	1,023 (20.6)	<0.001
Other‡	1 (0.2)	21 (1.8)	32 (1.4)	15 (1.4)	69 (1.4)	NS
Ear, nose, throat						
Otitis media	24 (5.1)	27 (2.3)	11 (0.5)	2 (0.2)	64 (1.3)	<0.001
Other‡	6 (1.3)	12 (1.0)	11 (0.5)	5 (0.5)	34 (0.7)	NS
Digestive tract						
Diarrhea/dehydration	3 (0.6)	26 (2.3)	30 (1.3)	19 (1.8)	78 (1.5)	NS
Liver or pancreas disorder	1 (0.2)	5 (0.4)	105 (4.6)	70 (6.6)	181 (3.6)	<0.001
Other‡	11 (2.3)	18 (1.6)	19 (0.8)	5 (0.5)	53 (1.1)	NS
Neurologic						
Encephalitis or myelitis	0	10 (1.1)	13 (0.6)	4 (0.4)	27 (0.5)	NS
Other‡	2 (0.4)	10 (0.9)	8 (0.3)	1 (0.1)	21 (0.4)	NT
Keratitis	0	1 (0.1)	9 (0.4)	6 (0.6)	16 (0.3)	
Other‡	10 (0.2)	21 (1.8)	32 (1.4)	17 (1.6)	80 (1.6)	NS
Death	0	2 (0.2)	6 (0.3)	2 (0.2)	10 (0.2)	NT

*Total excludes 12 patients with unknown date of birth. NS, not significant; NT, not tested (expected value <5). †By χ^2^ test. ‡Other complications (numbers in brackets indicate no. cases of that complication identified): pulmonary (e.g., bronchitis, pleurisy); ear, nose, throat (e.g., tonsillitis, sinusitis); digestive tract (e.g., vomiting, dysphagia, abdominal ache); neurologic (e.g., Guillain-Barré syndrome [1], meningitis); other (e.g., miscarriage [5], premature delivery [3], neonatal infection [4], myocarditis/pericarditis [8], general state impairment [42], thrombocytopenia [18]).

Neurologic complications included 1 case of myelitis and 26 cases of encephalitis (rate 0.6/1,000 cases). Of the encephalitis cases, 25 were acute disseminated encephalomyelitis, and 1 was measles-inclusion body encephalitis occurring 4 months after the initial appearance of measles rash. Patient male-female ratio was 0.8; the median age was 16 years (IQR 12–24 years).

Liver and/or pancreatic complications were reported in 5.0% of patients >15 years of age (p<0.001 for comparison of proportion of liver/pancreatic complications among patients >15 years of age and overall rate of liver/pancreatic complications). The proportion of hospitalized patients with otitis media (1.3%) varied significantly, from 5.1% for infants <1 year of age to <0.5% for patients >15 years of age (p<0.001).

Ten patients died (0.45 deaths/1,000 cases). Causes of death were pneumonia (7), encephalitis (1 acute disseminated encephalomyelitis, 1 measles-inclusion body encephalitis), and myocarditis (1). Nine of these patients were <30 years of age (median 23 years, range 11–68 years); 7 were female (male-female ratio 0.4). Seven patients were immunodeficient; 1 had congenital immunodeficiency and 6 had acquired immunodeficiency (e.g., Hodgkin’s lymphoma, Crohn’s disease, HIV, immunosuppressive treatment).

### Nosocomial Episodes

During the study period, 85 nosocomial episodes involving measles were reported; 73% occurred in emergency, internal medicine, and pediatric wards. These episodes involved 146 cases (most were also notified through mandatory reporting); 1 immunodeficient patient died. Twenty-five of these 85 episodes led to clusters with a median of 3 cases per episode (maximum 16); health care professionals were involved in 75% of those episodes.

### Circulating Genotypes

The measles virus genotype D5, predominant in 2008, stopped circulating in mid-2009 and was replaced by genotype D4. The latter accounted for 16.2% of 123 viruses genotyped in 2008, 75.0% of 284 genotyped in 2009, 97.2% of 696 genotyped in 2010, and 90.2% of 529 genotyped in 2011. The D4 genotypes are usually similar to the Montréal.CAN/89xD4 strain and drift from strain MVs/Enfield.GBR/14.07, which was first identified in Great Britain in 2007 (GenBank accession no. EF600554). The epidemic virus in France was the strain MVs/Montaigu.FRA/43.08, first identified during the second half of 2008, and was different from the MVs/Enfield.GBR/14.07, which was found in March 2008 during a nosocomial outbreak in Reims (Champagne-Ardenne region). Circulating to a lesser extent, other genotypes identified by the NRC were A, B3, D8, D9, H1, H2, and, recently, G3 (7.5% in 2011).

## Discussion

We describe an explosive outbreak of measles in France, with >22,000 cases reported during 2008–2011. Almost 5,000 persons were hospitalized, including >1,000 who had severe pneumonia and 27 who had encephalitis/myelitis. Ten persons died. As the virus spread nationwide, the most affected areas were, as expected, those with the lowest vaccine coverage, mainly in southern France. However, even districts with >90% MMR1 coverage in toddlers were affected, confirming that a very high level of immunity is required for measles elimination (*7*). Our data confirm that a shift in age at infection has occurred compared with that in the prevaccination era and that the risk for complications increases with age: half of the patients were >15 years of age, and among them, one third were hospitalized. In addition, incidence was highest among infants too young to be vaccinated.

Underreporting was estimated to be in excess of 50%, probably changing over time and with patient age. Local outbreak investigations found that <50% of cases had been notified, mainly because of secondary cases in households; these patients were less likely to seek medical advice once a first case had been diagnosed. Among the data collected from the main laboratories testing for measles IgM antibodies, the number of patients with measles-positive results was 1.5× higher than the number of notified cases. Cases diagnosed in hospitals were probably more often notified than those diagnosed in private practices (*8*); this may explain the higher proportions of hospitalized cases we observed, especially in adults with pneumonia, compared with those in the literature (*9*,*10*).

The absence of a vaccination registry in France precludes comprehensive documentation of patient vaccination status for most of the notified cases. For the majority of cases occurring in older children or young adults, patients did not bring along any vaccination documentation when seeking medical care for measles. Furthermore, these patients may have consulted a different practitioner than the one who followed them during their childhood. However, lack of documentation does not appear to have induced a substantial bias in the description of the vaccination status of the patients; vaccination status measured for patients who had written vaccination documentation (20.1%) was close to that measured in those without such documentation (17.4%). 

The proportion of vaccinated patients varied according to patient age, probably reflecting the disparity of vaccine coverage between age groups. Overall, 80% of notified cases occurred in unvaccinated persons. The geographic heterogeneity of vaccine coverage and virus exposure precluded the use of the screening method to estimate vaccine effectiveness (*11*). Nevertheless, the ease that the virus had in spreading could by no means be explained by lower than expected vaccine effectiveness. In 2011, the high number of 1-dose–vaccinated 20- to 29-year-old patients prompted the MOH to extend the 2-dose MMR schedule to everyone born since 1980 (*2*).

The NRC identified a D4 genotype variant as the predominant circulating strain during this outbreak. During the third wave, genotype G3 was identified; this genotype is known to have emerged in several other European countries and likely was imported from Southeast Asia (*12*).

Several European countries were affected by measles outbreaks during the same 4-year period as the outbreak period in France. The number of cases reported to the European Center for Disease Prevention and Control by the 29 participating countries increased 4-fold, from 7,817 in 2008 to 30,567 in 2011. Five countries (France, Italy, Romania, Spain, and Germany) accounted for >90% of all measles cases reported in 2011; France alone accounted for 50% (*13*–*15*). France also exported cases, not only to other European countries (*16*) but also to areas currently in the measles elimination certification process, such as the Americas (*17*), including the French districts of Martinique, Guadeloupe, and French-Guiana.

Our findings indicate that the measles epidemiologic profile observed in France in 2006–2007 was only a honeymoon period before reemergence, rather than an indication of imminent elimination. This reemergence was the consequence of persistent suboptimal vaccine coverage in toddlers and insufficient catch-up vaccination in older cohorts, leading to the growth of a large reservoir of susceptible persons.

A large outbreak had, in fact, been anticipated in France through modeling (*18*), analysis of coverage data (*19*), and a serosurveillance survey performed in 1998 (*20*). These findings led to initiatives to try to increase measles vaccination coverage. MMR mass media campaigns conducted from 1985 on were reinforced with promotional materials targeting vaccinators and the general public that were designed and distributed through various channels. In 1999, MMR vaccines became 100% free for children. Several studies aiming to identify barriers to MMR vaccination were conducted, and specific interventions to increase vaccine coverage were undertaken, particularly in low-performing districts. The studies consistently showed that the absence of MMR vaccination was the result of explicit parent and/or health care professional choice and not a lack of access to vaccination for geographic, financial, or sociocultural reasons (*21*,*22*). Consequently, tailor-made interventions were implemented, but their effect was disappointing (*21*); MMR1 coverage in children at 2 years of age did not increase above 90%.

To combat the reemergence of measles in France, more drastic control measures around sporadic cases and clusters were implemented in 2008–2009 by local health authorities. The main recommendations were to update the MMR vaccination status when needed and to propose postexposure vaccination or immunoglobulin injection (*1*). Media coverage of the epidemic emphasized the high likelihood of measles exposure associated with a risk for severe measles in young adults and in infants too young to be vaccinated, but these efforts were nevertheless unsuccessful in increasing coverage. Large catch-up vaccination campaigns in schools recommended by experts to the MOH were not implemented, primarily because of lingering effects from a hepatitis B vaccination scare that followed large school-based catch-up vaccination campaigns conducted in the 1990s (*23*). Furthermore, the controversy surrounding the large-scale influenza A(H1N1)pdm09 vaccination campaign conducted during 2009 likely further contributed to the MOH’s decision not to undertake any mass vaccination campaigns. In 2010, however, free measles vaccination was extended for children up to 17 years of age.

Although vaccine coverage improved over time during the outbreak, our experience confirms that high coverage in children is insufficient to avoid the spread of measles virus, especially when catch-up vaccination in older cohorts remains insufficient. Vaccine coverage figures at 2 years of age for children born in 2008 were 89.1% and 60.9% for MMR1 and MMR2, respectively, well below the respective national targets of 95% and 80%. Even if catch-up vaccination has resulted in vaccine coverage for adolescents reaching 95% and 84% for MMR1 and MMR2, respectively, the immunity level in young adults was still too low; results of a national seroprevalence survey conducted in 2009–2010 showed 9% of those 20–29 years of age were susceptible to measles (*24*).

These conclusions raise questions about the possibility of reaching elimination of highly communicable diseases for which levels of immunity >95% at an age as young as 2 years are required for elimination in societies in which a substantial proportion of the population is reluctant to vaccinate. Furthermore, it is necessary to reach and maintain these levels in each birth cohort to avoid new reservoirs of susceptible children. Regulation and social context in France do not currently favor mandatory immunization in the general population. Even more problematic, the very low levels of residual illness and death associated with this disease make effective communication about the serious risks involved difficult. 

In the first wave of this epidemic, attempts to convince health professionals and the general public about the urgent need to update the measles vaccination status of the target population were unsuccessful. Specific documents were prepared and widely disseminated, especially during European Immunization Week in 2009, 2010, and 2011, for which measles was chosen by the MOH as the national priority topic. Only in 2011, when the epidemic started to peak and when many hospitalizations, complications, and even deaths were highlighted, did sales data for MMR vaccines show an increase. At the same time, communication about the serious effects of the epidemic was reinforced and greater media coverage garnered. A mandatory check of measles immunization status, with reminders sent to parents of children who were not fully immunized, was also implemented in schools. Provisional results showed MMR1 coverage of 97%–98% in adolescents, which may have contributed to the absence of a notable fourth wave in 2012 (<500 cases reported through the end of June).

Will France be in a situation to meet the 2015 measles elimination target? A reservoir of susceptible persons certainly remains, but levels of susceptibility in those <20 years of age should now be close to the age-specific WHO elimination thresholds. The likelihood of future resurgence depends on several parameters that are difficult to document. The postepidemic level of seroprotection depends on the actual size of the 2008–2011 epidemic and the magnitude of the recent MMR vaccination catch-up for each dose in the various age groups. Clustering of the remaining susceptible persons still needs to be examined; several studies are planned or underway to document those parameters. Estimation of measles vaccination coverage at subdistrict level through a newly available exhaustive national vaccines reimbursement database will help to identify pockets of unvaccinated persons.

As useful as these studies might be, however, they will not solve the underlying issue of improved vaccination coverage through communication strategies targeting persons still reluctant to undertake MMR vaccination, either for themselves or for their children. It is likely that catch-up vaccination campaigns would have helped increase vaccine coverage, and although these are considered inappropriate in France at this time, such campaigns should be considered a primary tool in countries facing similar measles epidemic profiles.
